# COVID-19 Vaccination Acceptance During Pregnancy in Europe

**DOI:** 10.7759/cureus.63562

**Published:** 2024-07-01

**Authors:** Ioanna Tsiaousi, Alexandros Psarris, Marianna Theodora, Panagiotis Antsaklis, Michael Sindos, Pelopidas Koutroumanis, Dimitris Zacharakis, George Daskalakis

**Affiliations:** 1 1st Department of Obstetrics and Gynecology, National and Kapodistrian University of Athens School of Medicine, Athens, GRC; 2 1st Department of Obstetrics and Gynecology, General Hospital of Athens “Alexandra”, National and Kapodistrian University of Athens, Athens, GRC; 3 1st Department of Obstetrics and Gynecology, General Hospital of Athens "Alexandra" National and Kapodistrian University of Athens, Athens, GRC; 4 1st Department of Obstetrics and Gynecology, General Hospital of Athens “Alexandra”, National and Kapodistrian University of Athens, Athens, GRC, Athens, GRC; 5 1st Department of Obstetrics and Gynecology, National and Kapodistrian University of Athens Medical School, Athens, GRC

**Keywords:** covid, vaccination rates, pregnant women, covid-19 vaccination, acceptance

## Abstract

Pregnant women have been shown to have a higher risk of SARS-CoV-2 infection. Vaccination against the infection is the most effective strategy for preventing both severe disease and related complications. Nevertheless, vaccination hesitancy among pregnant women is an important issue affecting vaccine uptake and a major challenge for Public Health, as high rates of hesitancy can lead to complete refusal of vaccination, with health implications not only for the mother but also for the fetus. Based on the above, this review aims to capture the rates of vaccination against COVID-19 in pregnancy among European countries, from August 2020 to May 2022, as well as to highlight the predictive factors of its acceptance among pregnant women in these countries. The review of the available literature found that in Europe the acceptance of vaccination against COVID-19 among pregnant women varies with rates ranging from 21.3% to 87% for at least one dose and from 29.5% to 82.7%, for two doses of vaccine. Higher maternal education level, older age at pregnancy, previous vaccination against influenza and pertussis, positive attitude towards vaccines, and acceptance of vaccines during pregnancy are the most frequently reported positive predictors that are associated with higher vaccination rates. The information obtained from this study can contribute in the future, during epidemics or pandemics that may occur, to the development of targeted medical and communication strategies for the effective promotion of vaccination programs and the greatest possible coverage of the population, especially those belonging to vulnerable groups such as pregnant women.

## Introduction and background

Pregnant women represent a high-risk population for infectious respiratory diseases [1]. Changes in the immune system during pregnancy may increase the morbidity of infections, leading to higher complication rates and hospitalization compared to the general population. The COVID-19 pandemic has resulted in significant global death toll, creating public health challenges, overwhelming healthcare systems, disrupting supply chains, impacting the economy, and triggering a mental health crisis [2].

Pregnant women have been identified as having a higher risk of SARS-CoV-2 infection [3]. Although determining the exact number of incidents involving pregnant patients is challenging, current estimates suggest that over 20% women worldwide are of reproductive age, with approximately 5% pregnant at any given time. This implies millions of cases of COVID-19 in pregnant women over the past two years, making SARSCoV-2 infection one of the most widespread diseases in this population [4]. According to the Center for Disease Control and Prevention (CDC) in the United States, as of July 2022, 220,673 pregnant women had been diagnosed with COVID-19 infections [5].

COVID-19 vaccination is recognized as the most effective strategy against severe COVID-19 infection and related complications [6]. Numerous studies have shown that immunization during pregnancy is a safe and highly effective strategy, benefiting both the pregnant woman and the developing fetus [7]. However, vaccination programs' success depends on coverage, and vaccination hesitancy among pregnant women remains a significant issue affecting uptake.

Τhe present study aims to summarize COVID-19 vaccination rates in pregnancy across European countries, covering the period from August 2020 to May 2022.

## Review

Material and methods

A thorough search was conducted in the MEDLINE, Embase, CINAHL, and PubMed databases to identify English-language publications from September 2021 until December 2023. The search terms used included "vaccine acceptance", "willingness", "vaccination", "vaccine hesitancy", "pregnant women", "COVID-19 vaccine", "SARS-COV vaccine", and "uptake". Additional articles were manually retrieved from the reference lists of the papers. Only complete textual items were considered for the reference list.

COVID-19 vaccines and pregnancy 

The key competitors in the worldwide COVID-19 vaccine market, include virus-like particles (VLPs), subunit vaccines, DNA and RNA-based vaccines, viral vector-based vaccines, as well as inactivated and live-attenuated vaccines. Protein subunit, RNA-based and non-replicating viral vector-based platforms have been used mostly [8].

The exclusion of pregnant or nursing women from clinical trials has contributed to delayed recommendations [9,10]. Despite the lack of clinical trial results [9], professional organizations have acknowledged the need and globally recommend COVID-19 vaccination during pregnancy [11-14].

The mRNA vaccines BNT162b2 (Pfizer) and mRNA-1273 (Moderna) provided the first real evidence of safety and efficacy in the United States and Israel, the first countries to administer COVID-19 vaccinations during pregnancy [15].

Safety evaluation of COVID-19 vaccination during pregnancy

Safety concerns about COVID-19 immunization during pregnancy have been a major issue. Current research and systematic reviews support the recommendation that pregnant women should get vaccinated, as adverse reactions are not elevated [16].

Effectiveness of COVID-19 vaccines for pregnant women

In relation to the effectiveness of COVID-19 vaccines in expectant individuals, clinical data demonstrate comparable effectiveness between pregnant individuals and the general population [17]. Specifically, after 7-56 days following the second vaccination dose, the vaccine showed a 96% effectiveness rate and a 97% effectiveness rate against confirmed infection and symptomatic infection, respectively. The demographic and clinical characteristics of 10,861 vaccinated pregnant individuals aged 16 years or older without a history of SARS-CoV-2 were used to compare them to a control group of 10,861 pregnant women who had not received the vaccine [18]. In a case-control study involving 5,492 pregnancies, maternal vaccination, including supplemental doses, was linked to protection against complications associated with COVID-19 during pregnancy. mRNA COVID-19 vaccination of the mother, including a supplemental dose, resulted in protection against COVID-19 that required medical attention [19]. Another study found that the risk of SARS-CoV-2 infection and hospitalization due to COVID-19 were both reduced by 50% as a result of the vaccination against COVID-19 [20].

A national, population-based, historical cohort study of 82,659 and 33,303 pregnant women from the Delta and Omicron waves, was conducted in Israel to examine and compare the effectiveness of the third and second doses of the COVID-19 vaccine in preventing hospitalizations related to COVID-19 during pregnancy. The findings of this study indicate that administering a third dosage of the BNT162b2 mRNA COVID-19 vaccine, after a minimum interval of 5 months following the second dose, confers supplementary protection in pregnant individuals. Specifically, this additional dose reduces the likelihood of hospitalizations due to SARS-CoV-2 infection and mitigates the risk of experiencing major and severe disease [21].

The administration of COVID-19 vaccines during pregnancy provides protection not only to the pregnant individual but also to the neonate following delivery. Specifically, COVID-19 vaccination during pregnancy has been linked to a substantial reduction in the risk of perinatal adverse outcomes [22] and the prevention of neonatal COVID-19 intensive care unit (ICU) admissions [23].

Rates of acceptance and uptake of vaccines during pregnancy across European countries

Acceptance rates vary across European countries (Table 1). In Italy, up to 82.7% expressed an intention to get vaccinated [24-27], while active vaccination acceptance ranged from 21.3% to 79.9% [25,26,28,29]. Acceptance rates in Greece ranged from 53.9% [30] to 58.6% [31], while in Turkey it reached 37.7% [32]. In Spain, the intention to get vaccinated was found to be 67% in the pregnant population, but finally, a 56% acceptance rate was reported [33]. In France, vaccination acceptability was found to be 65.8%, including women who had a minimum of one administration of the COVID-19 vaccine [34]. Similarly, 74% of expectant mothers in Poland, including those who received a follow-up dose of the vaccine during the course of their pregnancy, accepted the vaccination [35]. In Romania, 53% of expectant mothers had received vaccinations [36] while 52.2% of them were hesitant about vaccination [37]. The COVID-19 vaccine was widely accepted by pregnant and nursing women in the Czech Republic (70.2%); however, there was a notable variation between the two groups (76.6% and 48.8% respectively) [38]. Germany reported 47.2% and 39.5% acceptance rates in pregnant and lactating women, respectively [39]. Sweden and Norway have acceptance rates in the pregnant population of 78% and 87%, respectively [40]. In the United Kingdom, vaccine acceptance during pregnancy ranged between 62.1% [41] and 69% [42], while in Ireland it was 38% [43]. In Switzerland, the percentage of pregnant and lactating women who reported the intention to get vaccinated during the first year of the pandemic was 29.7% and 38.6%, respectively [44].

**Table 1:** Rates of acceptance and uptake of vaccines during pregnancy

Authors and year of publication	Aim of the study	Country	Sample	COVID-19 vaccination rate- COVID-19 vaccine acceptance rate among pregnant women
Maranto et al. 2023 [24]	The identification of the factors associated with vaccine adherence in a group of women suffering high-risk pregnancies.	Italy	233 pregnant women	65.2% of the enrolled women indicated a willingness to be vaccinated, and 46.8% had already received the anti-SARS-CoV-2 vaccination.
Miraglia Del Giudice et al. 2022 [25]	The evaluation of the uptake, the hesitancy, and the willingness regarding the vaccination against the SARS-CoV-2	Italy	385 women	21.3% of the participants were vaccinated when pregnant 71.9% were willing to be vaccinated
Lubrano et al 2022 [26]	To estimate maternal COVID-19 vaccine uptake as well as vaccination barriers in the Lombardy region	Italy	926 women	22.7% of participants had received at least one vaccine dose
Colciago et al. 2022 [27]	To investigate expectant women's attitudes and risk perceptions regarding the COVID-19 vaccine, as well as the reasons for their hesitancy to receive the vaccine.	Italy	538 women	82.7% of pregnant women accepted COVID-19 vaccination
Cetin et al. 2022 [28]	To describe SARS-CoV-2 vaccination rates among pregnant women, to compare vaccination rates among pregnant women and women of reproductive age, and to assess the impact of vaccination status among pregnant women on admissions to intensive care units in 2021.	Italy	122,942 pregnant women	79.9% of the pregnant women had received the first dose during pregnancy by the end of December 2021.
Galanis et al. 2023 [29]	The comprehension of the factors linked to the uptake of the COVID-19 vaccine among pregnant women.	Greece	812 pregnant women	58.6% of pregnant women had received a COVID-19 vaccine.
Tsiaousi et al. 2023 [30]	To examine the vaccination acceptance of COVID-19 vaccine in the Hellenic pregnant population and to conduct a comparative analysis of the factors that influence the decision to receive the vaccine during pregnancy.	Greece	800 pregnant women	The rate of vaccine coverage was 53.9%.
Karagöz Özen et al. 2022 [31]	To examine the immunization rates among pregnant women as well as explore the factors contributing to vaccine reluctance within this particular patient population.	Turkey	247 pregnant women	37.7% of the 247 pregnant women had received the COVID-19 vaccine during pregnancy.
Goncu Ayhan et al. 2021 [32]	To evaluate pregnant women's acceptance and reluctance toward COVID-19 vaccines.	Turkey	300 pregnant women	37% of expectant women indicated they would receive the vaccine if it were recommended for pregnant women.
Huré et al. 2022 [33]	To assess the achievement of COVID-19 vaccination and variables related with women during pregnancy in the postpartum period	France	371 women	65.8% had received at least one dose of COVID-19 vaccine during pregnancy.
Egloff et al. 2022 [34]	To evaluate the perceptions and acceptance of COVID-19 vaccination among pregnant women.	France	664 pregnant women	29.5% of pregnant women would agree to be vaccinated against COVID-19.
Örtqvist et al. 2022 [35]	To examine the background factors that are correlated with individuals who have not received vaccinations.	Sweden and Norway	164560 women	In Sweden, approximately 78% of the studied population has received at least one dose of the vaccine upon delivery, while in Norway, the rate is 87%.
Citu et al. 2022 [36]	To determine the level of adoption of the COVID-19 vaccination campaign among pregnant women in Romania, as well as the factors that influence their decisions.	Romania	345 pregnant women	The vaccination adoption rate was 53%
Citu et al. 2022 [13]	To examine the prevalence and extent of COVID-19 vaccine reluctance among expectant women in Romania, as well as the factors that influence their decision	Romania	184 pregnant women	The rate of hesitancy about COVID-19 vaccination was 52.2%.
Riad et al. 2021 [37]	Το assess Czech pregnant and breastfeeding women's opinions toward COVID-19 vaccinations and the factors influencing their attitudes	Czechia	362 pregnant and lactating women	The total COVID-19 vaccination acceptability 70.2%, with a significant difference between pregnant (76.6%) and nursing (48.8%) women.
Nowacka et al. 2022 [38]	To determine the vaccination adoption and hesitancy rate, characteristics, and decision-making factors among pregnant and postpartum women.	Poland	1033 pregnant (54.1%) and postpartum (45.9%) women	The vaccination adoption rate was 74%
Marbán-Castro et al. 2022 [39]	To examine the views of pregnant women and healthcare workers (HCWs) towards the COVID-19 vaccination.	Spain	302 pregnant women and 309 HCWs	67% of pregnant participants indicated they would accept receiving vaccinations during current pregnancy 56% of pregnant participants were vaccinated against COVID-19 during current pregnancy
Skirrow et al. 2022 [40]	To investigate expectant women's perspectives on the acceptability of the COVID-19 vaccine for themselves while pregnant, when not pregnant, and for their unborn children.	UK	1181 women	COVID-19 vaccine acceptance rate during pregnancy was 62.1%.
Mhereeg et al. 2022 [41]	to assess the prevalence of COVID-19 immunization among pregnant women in Wales and examine its correlation with factors such as age, ethnicity, and socioeconomic status, utilizing electronic health record data linkage. Additionally, the study seeks to investigate the perspectives of pregnant women towards the administration of the COVID-19 vaccine during pregnancy.	UK	25,111 pregnant women	69% of the individuals expressed their willingness to receive the vaccine while being pregnant. 32.7% received at least one dose of the COVID-19 vaccine during pregnancy, 34.1% remained unvaccinated throughout the study period, and 33.2 percent was given the vaccine postpartum.
Geoghegan et al. 2021 [42]	To investigate expectant women's attitudes toward COVID-19 vaccines	Ireland	300 pregnant women	COVID-19 vaccine acceptance rate during pregnancy was 38%.
Schaal et al. 2022 [43]	Τo investigate pregnant and nursing women's attitudes toward COVID-19 vaccination.	Germany	2339 women (n = 1043 pregnant and n = 1296 breastfeeding)	47.2% of pregnant women and 39.5% of lactating women were willing to be vaccinated
Stuckelberger et al. 2021 [44]	To evaluate the willingness of expectant and nursing Swiss women to become vaccinated	Switzerland	1551 women	29.7% of pregnant and 38.6% of breastfeeding women expressed willingness to get vaccinated against COVID-19 if a vaccine had been available during the first wave.

Predictors of maternal willingness to receive vaccination and barriers to COVID-19 vaccination among pregnant women across European countries

In Italy, a higher educational level [24,26,28], active work status, comorbidities [26], a history of COVID-19 infection before pregnancy [25], pertussis vaccination history [27], official recommendations by Health Authorities [28], low perception of barriers to immunization [24], as well as recommendations by health care professionals, particularly gynecologists [24-26], who serve as the primary source of information, have emerged as significant factors associated with the acceptance of SARS-CoV-2 vaccination among pregnant women [24]. Conversely, the absence of recommendations from Healthcare professionals [26] and concerns about the vaccine's safety during pregnancy [26,27] were identified as major factors negatively impacting vaccination acceptance. Additionally, vaccination hesitancy was associated with a history of COVID-19 infection during pregnancy [27] and reliance on information from the mass media, internet, and social networks [25]. See Table 2 for a summary.

**Table 2:** Predictors of maternal willingness to receive vaccination and barriers to COVID-19 vaccination among pregnant women across European countries.

Authors and year of publication	Aim of the study	Country	Sample	Predictors of maternal willingness towards receiving vaccination	Barriers to COVID-19 vaccination among pregnant women
Maranto et al. 2023 [24]	The identification of the factors associated with vaccine adherence in a group of women suffering high-risk pregnancies.	Italy	233 pregnant women	Higher educational level Obtaining information from gynecologists A low level of perceived barriers to vaccination	Trusting mass media/internet/social networks for information
Miraglia Del Giudice et al. 2022 [25]	The evaluation of the uptake, the hesitancy, and the willingness regarding the vaccination against the SARS-CoV-2	Italy	385 women	Higher educational level Having at least one relative/cohabitant partner/friend who had been infected by SARS-CoV-2 Higher perceived concern of being infected Obtaining information about the vaccination from gynecologists No need for additional information about vaccination against COVID-19 History of infection by SARS-CoV-2 before pregnancy	Trusting mass media/internet/social networks for information Concerns about safety and efficacy of the vaccines Lack of knowledge
Lubrano et al 2022 [26]	To estimate maternal COVID-19 vaccine uptake as well as vaccination barriers in the Lombardy region	Italy	926 women	High and middle education level Employment Presence of comorbidities	Safety concerns The individual did not obtain any recommendation from a healthcare provider
Colciago et al. 2022 [27]	To investigate expectant women’s attitudes and risk perceptions regarding the COVID-19 vaccine, as well as the reasons for their hesitancy to receive the vaccine.	Italy	538 women	Previous vaccination against pertussis	History of COVID-19 infection during pregnancy Lack of safety data in pregnancy and the possibility of harm to the fetus
Cetin et al. 2022 [28]	To describe SARS-CoV-2 vaccination rates among pregnant women, to compare vaccination rates among pregnant women and women of reproductive age, and to assess the impact of vaccination status among pregnant women on admissions to intensive care units in 2021.	Italy	122,942 pregnant women	Higher education level The announcement of official recommendations by institutions Italian citizenship Older maternal age	
Galanis et al. 2023 [29]	The comprehension of the factors linked to the uptake of the COVID-19 vaccine among pregnant women.	Greece	812 pregnant women	Increased danger, contamination fears, and fears about economic consequences Higher levels of trust in COVID-19 vaccines. Older age Perceived health status as “poor” Τhe absence of a prior diagnosis of COVID-19	Uncertainties regarding the safety and efficacy of COVID-19 vaccines. Fear that COVID-19 vaccines may damage their unborn child. Concern for adverse effects. Stress caused by the compulsive checking and reassurance seeking
Tsiaousi et al. 2023 [30]	To examine the vaccination acceptance of COVID-19 vaccine in the Hellenic pregnant population and to conduct a comparative analysis of the factors that influence the decision to receive the vaccine during pregnancy.	Greece	800 pregnant women	Employment Older age Higher monthly income Vaccination against influenza/pertussis during pregnancy Adequate knowledge of vaccines Health professionals' and scientific sites' information	
Sezerol and Davun 2023 [45]	To assess the vaccination hesitancy among pregnant women who declined to receive the COVID-19 vaccine during the pandemic era, as well as to identify the potential variables influencing their decision-making process.	Turkey	561 pregnant women	Recommendations for vaccination by health professionals	Higher level of education Higher income Fear of vaccination side effects Belief in the ineffectiveness of the vaccine Convictions that the vaccine is unnecessary Not finding enough time to be vaccinated Beliefs in the existence of a conspiracy theory, not believing in the pandemic. Religious beliefs
Karagöz Özen et al. 2022 [31]	To examine the immunization rates among pregnant women as well as explore the factors contributing to vaccine reluctance within this particular patient population.	Turkey	247 pregnant women		Fear of vaccination side effects Vaccination before pregnancy
Goncu Ayhan et al. 2021 [32]	To evaluate pregnant women's acceptance and reluctance toward COVID-19 vaccines.	Turkey	300 pregnant women	First trimester of pregnancy	Absence of data on the safety of the COVID-19 vaccine for expectant populations Fear of vaccination side effects on the fetus Second and third trimester of pregnancy
Huré et al. 2022 [33]	To assess the achievement of COVID-19 vaccination and variables related with women during pregnancy in the postpartum period	France	371 women	Older age Higher socio-professional category Prior knowledge offered by medical practitioners	Fear of vaccination adverse effects Negative attitude towards vaccines in general
Egloff et al. 2022 [34]	To evaluate the perceptions and acceptance of COVID-19 vaccination among pregnant women.	France	664 pregnant women	Being slightly older Multiparity Having discussed vaccination with a caregiver Acceptance of the influenza vaccine	Fear of vaccination adverse effects
Örtqvist et al. 2022 [35]	To examine the background factors that are correlated with individuals who have not received vaccinations.	Sweden and Norway	164560 women	Higher income levels Having pre-pregnancy comorbidities Having preeclampsia	Age ≤30 years old and above 40 years old (only in Norway) Mothers having multiple births Lower educational level Being born outside the Scandinavian countries Smoking during pregnancy Living alone Having gestational diabetes Abnormal body mass index
Nowacka et al. 2022 [38]	To determine the vaccination adoption and hesitancy rate, characteristics, and decision-making factors among pregnant and postpartum women.	Poland	1033 pregnant and postpartum women	Νulliparity Ηigher education Ηistory of COVID-19 infection Α vaccination offer made by a physician or midwife Positive attitude of a physician or midwife toward immunization Information provided by a physician or midwife regarding the elevated risk of COVID-19 complications	Having children Lower educational status
Citu et al. 2022 [36]	To determine the level of adoption of the COVID-19 vaccination campaign among pregnant women in Romania, as well as the factors that influence their decisions.	Romania	345 pregnant women	Urban area of residence Higher education level Third trimester of pregnancy Having trust in the government Being a frequent traveler Fearing the severity of COVID-19 Greater local availability of COVID-19 vaccines Observing an increase in vaccination rates	
Citu et al. 2022 [13]	To examine the prevalence and extent of COVID-19 vaccine reluctance among expectant women in Romania, as well as the factors that influence their decision	Romania	184 pregnant women		Βeing unafraid of COVID-19 Lower-than-average income Believing rumors on social media Not believing in SARS-CoV-2 existence Being a vaccination non-believer
Riad et al. 2021 [37]	Τo assess Czech pregnant and breastfeeding women's opinions toward COVID-19 vaccinations and the factors influencing their attitudes	Czechia	362 pregnant and lactating women	Third trimester of pregnancy Older age Higher education level Trust in industry and healthcare professionals	Having previous live births
Marbán-Castro et al. 2022 [39]	To examine the views of pregnant women and healthcare workers (HCWs) towards the COVID-19 vaccination.	Spain	302 pregnant women and 309 HCWs	Recommendation from an HCW Individuals wish to safeguard themselves and their infants from the occurrence of illness.	Concern about potential harm to the mother and the child The absence of access to the COVID-19 vaccination
Skirrow et al. 2022 [40]	To investigate expectant women's perspectives on the acceptability of the COVID-19 vaccine for themselves while pregnant, when not pregnant, and for their unborn children.	UK	1181 women	Trust in vaccines Trust in the health system	Lower-income households Age under 25-years old Not being vaccinated against pertussis in pregnancy Safety concerns about COVID-19 vaccines
Mhereeg et al. 2022 [41]	to assess the prevalence of COVID-19 immunization among pregnant women in Wales and examine its correlation with factors such as age, ethnicity, and socioeconomic status, utilizing electronic health record data linkage. Additionally, the study seeks to investigate the perspectives of pregnant women towards the administration of the COVID-19 vaccine during pregnancy.	UK	25,111 pregnant women	Belonging to Asian and other ethnic groups	Younger age (<30 years) Living in areas of high deprivation Belonging to White groups Concerns regarding the dearth of research on the baby's long-term outcomes Anxiety about vaccines Inconsistent advice/information
Geoghegan et al. 2021 [42]	To investigate expectant women's attitudes toward COVID-19 vaccines	Ireland	300 pregnant women	Higher education levels Visiting private or semi-private clinic Age 30-35 years old Gestational age greater than 31 weeks Recommendation from obstetrician, GP or public health officials	Concerns about unknown long-term effects Concerns about how new the vaccine was
Schaal et al. 2022 [43]	Τo investigate pregnant and nursing women's attitudes toward COVID-19 vaccination.	Germany	2339 women (n = 1043 pregnant and n = 1296 breastfeeding)	Fear of infection and the development of disease symptoms	Lack of information Lack of scientific evidence regarding vaccine safety . Worries about risk of harm to the baby
Stuckelberger et al. 2021 [44]	To evaluate the willingness of expectant and nursing Swiss women to become vaccinated	Switzerland	1551 women	Maternal age >40 years Higher educational level Vaccination against influenza within the past year Having an obstetrician as the primary healthcare provider Τhird trimester of pregnancy Having a positive diagnosis of SARS-CoV-2 Living with someone older than 65 years old	Being German-speaking Being in their second trimester of pregnancy

In Greece, primary reasons for non-vaccination during pregnancy included concerns about the safety and effectiveness of COVID-19 vaccines, fear of potential harm to the fetus, and apprehensions about long-term side effects. Conversely, positive drivers for COVID-19 vaccine acceptance in pregnancy encompassed fears of COVID-19 severity, concerns about financial impact [29], a high acceptance of pediatric vaccines, vaccination against influenza during the current or previous pregnancy, and vaccination against pertussis during the current pregnancy [30]. Sociodemographic data indicated a significant and positive correlation between vaccine acceptability and employment, higher maternal age, and greater monthly income [30].

In Spain, vaccine acceptance was strongly associated with healthcare professionals' (HCPs') recommendations and the mother’s desire to provide optimal protection to the fetus. Conversely, barriers to maternal vaccination for COVID-19 included safety concerns about the vaccines [39].

In France, drivers of vaccination acceptance during pregnancy comprised of older maternal age [33,34], higher socio-occupational class [33], multiparity [34], acceptance of influenza vaccine during pregnancy [34], consultations provided by HCPs [33,34], and the perception that vaccination could offer protection to the fetus [33]. On the other hand, major barriers were the fear of safety issues and negative perceptions of vaccines in general [33].

Among Czech pregnant and lactating women, a higher acceptance rate was associated with older maternal age, a higher level of education, first pregnancy, and trust in health professional consultation [37]. In Sweden and Norway, COVID-19 vaccination hesitancy was associated with maternal age under 30 and over 40 (in Norway), multiparity, lower education level, birth outside of Scandinavia, smoking during pregnancy, living alone, and having gestational diabetes. In contrast, vaccination rates were higher among pregnant women with higher income, pre-pregnancy comorbidities, and pre-eclampsia. Additionally, in Sweden, women with lower and higher BMI compared to those of normal body weight were more reluctant to get vaccinated, while in Norway, similar findings were observed only for pregnant women with lower BMI [35].

In Poland, the main drivers of COVID-19 vaccination included younger age, higher education level, previous COVID-19 infection, administration of vaccination by a physician or midwife and supportive consultation, including information about the severity of the disease. Conversely, the main barriers included the presence of children, lower educational levels, and the absence of past COVID-19 infection [38].

In Romania, statistically significant variables affecting the vaccination acceptance rate included living in an urban area, having a higher level of education, being pregnant in the third trimester, trusting the government, frequent travel, fear of the severity of the illness, having access to vaccines, and a high level of vaccination acceptance in the general population [36].

In Turkey, vaccination hesitancy in the pregnant population was associated with higher educational levels and higher income. Conversely, positive HCP recommendations had the opposite impact [45]. Vaccination hesitancy was also associated with the lack of safety and efficacy data on the COVID-19 vaccine during pregnancy [32], fear of vaccine side effects [31,32,45], and pre-pregnancy vaccination [31].

Studies from the United Kingdom found that younger maternal age [40,41], residing in areas of high deprivation [41], low family income, non-vaccination against pertussis during pregnancy, concern about vaccine safety [40] and absence of research on the long-term effects of the vaccine on the baby, fear of vaccines in general, inconsistent advice/information [41] were among the factors associated with non-acceptance of vaccination [40], as opposed to trusting in vaccination in general and towards the health system being associated with acceptance of vaccination in this population group [40]. Also, Asians and other ethnic groups were more likely to receive the vaccine than those in white groups [41]. In Ireland, higher levels of education, being monitored at a private or semi-private clinic, recommendation of vaccination by health professionals, maternal age of 30-35 years, and gestational age of more than 31 weeks were associated with greater acceptance of vaccination among pregnant women. Conversely, concern about the possible long-term effects of the vaccine and how new the vaccine was acted as deterrents to its acceptance [42].

In Germany, the reasons for not accepting the vaccine included insufficient information, lack of scientific data on the safety of the vaccine, and concern about the possible harmful effects of the vaccine on the fetus. Conversely, fear of infection and developing disease symptoms were associated with willingness to vaccinate among pregnant and lactating women [43].

In Switzerland, maternal age over 40 years, higher educational level, history of influenza vaccination during the previous year, having an obstetrician as a primary health care professional, third trimester of pregnancy, diagnosis with SARS-CoV- 2 as well as cohabitation with an elderly person over 65 years of age were positively associated with vaccine acceptance among pregnant women [44].

Discussion

Vaccination hesitancy during pregnancy poses a significant challenge to Public Health, as unchecked hesitancy may lead to complete refusal of immunization, thereby impacting maternal and fetal health.

The primary objective of this study was to elucidate the acceptance rates of COVID-19 vaccination during pregnancy across European countries, examining the drivers and barriers to vaccination. This information is crucial for the development of targeted medical and communication strategies [2]. Across Europe, the acceptance of COVID-19 vaccination in pregnancy varied widely, ranging from 21.3% to 87% for at least one dose and from 29.5% to 82.7%, for two doses. Notably, Italy reported a relatively high vaccination acceptance rate among pregnant women [24,25,27,28]. Conversely, other European countries including Romania [13,36], Ireland [42], Germany [43] and Switzerland [44] recorded lower rates of vaccination and acceptance often below 50%. Interestingly, countries severely impacted by the pandemic, such as Italy, demonstrated a notably high level of acceptance among pregnant women from the early stages of the pandemic [2].

The most frequently cited positive prognostic factors associated with higher vaccination rates include higher maternal educational level [24-26,28,35-38,42,44], older maternal age during pregnancy [28-30,33,34,37,40-42,44], previous vaccination against pertussis and influenza [27,30,40,44], and a positive attitude towards vaccines and acceptance of vaccines during pregnancy [13,33,34,40].

Research indicates that women with higher education status were 1.99 times more likely (95% CI: 1.25-3.19) to receive immunization compared to those with a secondary level education or below [46]. Additionally, individuals with a Master's or doctoral degree exhibited a 5.99-fold higher intention to receive a COVID-19 vaccination dosage (OR: 5.99; 95% CI: 1.12-32.16) [47]. Studies have shown that a high educational level is associated with better financial status, a superior understanding of scientific information, and improved access to the health system and healthcare professionals [24]. Despite this, one Turkish study [45] demonstrated that a higher educational level of pregnant women was associated with a lower acceptance rate, suggesting a need for more safety data to accept the COVID-19 vaccine [48].

Older maternal age during pregnancy is consistently reported as a factor associated with an increased likelihood of accepting and receiving the COVID-19 vaccine [28-30,33,34,37,40-42,44]. A study conducted in the United Kingdom found that individuals in the 40-50 age range were 1.33 times more likely to get vaccinated than those in the 25-29 age range (HR = 1.33, 95% CI 1.18 to 1.49, p < 0.001). Similarly, those aged 25-29 were 1.17 times less likely to have the vaccination compared to those aged 30-39 [41]. This finding may be explained by the increased fear of COVID-19 among older pregnant women. 

Another significant factor influencing acceptance was previous maternal vaccinations against influenza and pertussis, along with a positive attitude towards vaccines in general [27,30,33,34,40,44]. Studies have shown that pregnant individuals who were not vaccinated against influenza and pertussis were respectively three and four times more likely to refuse the COVID-19 vaccine during pregnancy [27]. Conversely, there was a 365% increased likelihood of receiving the COVID-19 vaccine for expectant mothers (B = 1.54 OR = 4.65, p < 0.01) [49]. Furthermore, research has demonstrated that those who expressed positivity to COVID-19 vaccination in general had a 4.81 times higher chance of being vaccinated (AOR: 4.81, 95% CI: 1.42-7.33) [50].

Active employment status has been consistently linked to higher acceptance of vaccination against COVID-19 [26,30]. According to research by Sznajder et al. [51], pregnant women with full-time jobs were twice as likely to be willing to get the COVID-19 immunization (AOR: 2.22; 95% CI: 1.02, 4.81).

Low income has been associated with greater hesitancy and resistance of pregnant women towards vaccination [13,30,35,40], as well as lower socio-professional status [33]. One study found that women with household incomes less than £25,000 were three times more likely than those in the highest income category (>£80,000; 9.4%) to categorically refuse the COVID-19 vaccination during pregnancy (28%) [40]. Furthermore, separate research revealed that those with a monthly salary beyond the average were 1.13 times more reluctant, but those with a lower income were 2.52 times more hesitant [45].

Our review indicates that data related to prior COVID-19 infection and acceptance were inconsistent in pregnant women [25,27,29,38].

The absence of other children was also associated with the reluctance of pregnant women to receive vaccination [34].

Increased understanding about immunizations during pregnancy was shown to reduce hesitancy 3.88 times (OR: 3.88, 95% CI: 2.37-6.35, p-value < 0.000001) [52]. This could potentially account for the observed results in Poland [38], Czechia [37], Sweden, and Norway [35], where having previous live births was linked to higher resistance towards vaccination. However, one study discovered that having no children was linked to a 1.74-fold higher chance of accepting vaccines (OR: 1.74; 95% CI: 1.43-2.12) while having more than one child was linked to a 4.76-fold higher chance of not wanting to get vaccinated (≥3, OR: 4.76, p = 0.006) [38].

The desire to protect loved ones, particularly those belonging to vulnerable groups such as the elderly, also emerged among the factors associated with vaccine acceptance among pregnant women in Switzerland [44].

Having an underlying condition or a high-risk pregnancy was related to a higher acceptance rate [26,35,29]. According to Khan et al. [46], pregnant women with comorbidities had a 3.72-fold higher chance (95% CI: 1.57-8.83) to accept COVID-19 vaccine compared with pregnant women without comorbidities. In contrast, according to another study, pregnant women without pregnancy-related health conditions were found to be 4.131 times less likely to accept the COVID-19 vaccination [53].

On the other hand, Bagalb et al. [54] demonstrated that pregnant women who believe they are young and healthy are three times more likely to refuse COVID-19 vaccination.

In the present study, refusal of vaccination among pregnant women was also associated with gestational age, with older gestational age being associated with reluctance of pregnant women to be vaccinated in Turkey [32], or acceptance of vaccination in European countries (Romania, Czech Republic, Switzerland, and Ireland) [36,37,42,44]. Based on research, the likelihood of accepting the vaccination during the third trimester was 6.501 times higher (95% CI: 1.207-35.030) compared to the first trimester [37].

Understanding how pregnant women receive information about vaccination is extremely important, given the association of specific sources with different levels of knowledge, the high availability of misleading information, and the positive effect of information received from health professionals regarding the choices related to their vaccination [55]. Specifically, the extensive use of the internet and social media has made it more challenging to counteract the notable rise in vaccination hesitancy and the anti-vaccination movement throughout the last 20 years. Furthermore, the Internet and social media offer more channels for vaccine disinformation and can sway those who are reluctant about vaccinations in today's technologically aware society. By disseminating false information to discourage vaccination, people who are vaccine-hesitant or moderately resistant may be greatly impacted by increased access to technology, particularly social media [56].

The above could possibly explain the higher rates of increased hesitancy of COVID-19 vaccination among pregnant women who trusted the Internet and social networks for information about vaccines and the disease [13,24,25]. On the other hand, receiving relevant information and vaccination recommendations from the gynecologist and, in general, from health professionals [24,25,30,33,34,39,42,45] was associated with greater acceptance of vaccination. According to a recent meta-analysis, Strong Recommendations by HCPs during the 2009 influenza pandemic increased by six times the acceptance of the influenza vaccine. In another study, 52% of participants received the COVID-19 vaccine provided that there was a recommendation by an HCP (aPR: 1.52; 95 % CI: 1.31, 1.76) [57].

The fast-track procedures employed in the development and regulatory processes of COVID-19 vaccines have had a controversial impact, particularly regarding safety and effectiveness. The exclusion of pregnant women from clinical trials is strongly associated with existing hesitancy, primarily rooted in safety concerns. According to Regan et al. [57], vaccination acceptance was 2-3 times higher in cases where safety data were available (aPR: 2.86; 95% CI: 2.49, 3.29). Similarly, acceptance of the COVID-19 vaccination was 2.54 times higher if available data were evaluated as having sufficient safety and efficacy testing (aPR: 2.54; 95% CI: 2.25, 2.86). Moreover, research indicates that pregnant women receiving comprehensive information regarding COVID-19 disease and the COVID-19 vaccine are almost 2.5 times more likely to consent to immunization. Information provided by Governmental Official Health Bodies also had a positive impact on vaccination acceptance [58]. These data emphasize the need to provide more safety data to pregnant women to increase trust and create resistance to fake news and misinformation. Simultaneously, it is crucial for HCPs to disseminate information about the potential risk of COVID-19 infection to enhance patients' self-confidence and risk assessment - two critical determinants of vaccine hesitancy [59].

Furthermore, research has revealed that confidence in business and healthcare experts has a substantial impact on vaccination acceptance. Specifically, faith in the industry increases the acceptance odds ratio (AOR) by 15.590 times (95% CI: 1.754-138.599), while trust in healthcare professionals increases it by 4.355 times (95% CI: 1.277-14.847) compared to their counterparts [37]. Overall, research has demonstrated that people's readiness to accept public health solutions in times of crisis is influenced by their level of faith in the local public health administration. The willingness of people to adopt public health measures to control disease outbreaks during the COVID-19 pandemic depends on their level of trust in the local response to public health emergencies [60].

## Conclusions

This comprehensive review of international literature underscores the complexity of vaccine acceptance among pregnant women, influenced by a myriad of factors, including psychosocial, demographic, cultural, and health system-related factors, among others. Across countries, regardless of income level, sources of information consistently emerged as a dominant factor influencing personal beliefs and attitudes. Safety and efficacy concerns were common issues reported in most regions.

A thorough exploration and understanding of vaccine hesitancy and its related factors, coupled with the education of healthcare professionals on these findings, are deemed crucial for achieving optimal vaccination acceptance, coverage, and ultimately, herd immunity. These insights can contribute significantly to the development of effective, multilevel actions and interventions, tailored to targeted populations. By addressing the identified reasons for vaccine hesitancy among pregnant women, these findings offer guidance to future situations ensuring a focused and informed approach to boost vaccine acceptance, particularly within the vulnerable population of pregnant women.
